# Innate immune responses induced by lipopolysaccharide and lipoteichoic acid in primary goat mammary epithelial cells

**DOI:** 10.1186/s40104-017-0162-8

**Published:** 2017-04-01

**Authors:** Omar Bulgari, Xianwen Dong, Alfred L. Roca, Anna M. Caroli, Juan J. Loor

**Affiliations:** 1 Department of Animal Sciences and Division of Nutritional Sciences, University of Illinois at Urbana-Champaign, Urbana, IL 61801 USA; 2 Department of Molecular and Translational Medicine, University of Brescia, Brescia, 25123 Italy; 3 Institute of Animal Nutrition, Sichuan Agricultural University, Chengdu, 611130 China; 4 Division of Nutritional Sciences, University of Illinois at Urbana-Champaign, Urbana, IL 61801 USA

**Keywords:** Gene expression, Inflammation, Lactation, Mastitis

## Abstract

**Background:**

Innate immune responses induced by in vitro stimulation of primary mammary epithelial cells (MEC) using Gram-negative lipopolysaccharide (LPS) and Gram-positive lipoteichoic acid (LTA) bacterial cell wall components are well﻿- characterized in bovine species. The objective of the current study was to characterize the downstream regulation of the inflammatory response induced by Toll-like receptors in primary goat MEC (pgMEC). We performed quantitative real-time RT-PCR (qPCR) to measure mRNA levels of 9 genes involved in transcriptional regulation or antibacterial activity: Toll-like receptor 2 (*TLR2*), Toll-like receptor 4 (*TLR4*), prostaglandin-endoperoxide synthase 2 (*PTGS2*), interferon induced protein with tetratricopeptide repeats 3 (*IFIT3*), interferon regulatory factor 3 (*IRF3*), myeloid differentiation primary response 88 (*MYD88*), nuclear factor of kappa light polypeptide gene enhancer in B-cells 1 (*NFKB1*), Toll interacting protein (*TOLLIP*), and lactoferrin (*LTF*). Furthermore, we analyzed 7 cytokines involved in Toll-like receptor signaling pathways: C-C motif chemokine ligand 2 (*CCL2*), C-C motif chemokine ligand 5 (*CCL5*), C-X-C motif chemokine ligand 6 (*CXCL6*), interleukin 8 (*CXCL8*), interleukin 1 beta (*IL1B*), interleukin 6 (*IL6*), and tumor necrosis factor alpha (*TNF*).

**Results:**

Stimulation of pgMEC with LPS for 3 h led to an increase in expression of *CCL2*, *CXCL6*, *IL6*, *CXCL8*, *PTGS2*, *IFIT3*, *MYD88*, *NFKB1*, and *TLR4* (*P* < 0.05). Except for *IL6*, and *PTGS2*, the same genes had greater expression than controls at 6 h post-LPS (*P* < 0.05). Expression of *CCL5*, *PTGS2*, *IFIT3*, *NFKB1*, *TLR4*, and *TOLLIP* was greater than controls after 3 h of incubation with LTA (*P* < 0.05). Compared to controls, stimulation with LTA for 6 h led to greater expression of *PTGS2*, *IFIT3*, *NFKB1*, and *TOLLIP* (*P* < 0.05) whereas the expression of *CXCL6*, *CXCL8*, and *TLR4* was lower (*P* < 0.05). At 3 h incubation with both toxins compared to controls a greater expression (*P* < 0.05) of *CCL2*, *CCL5*, *CXCL6*, *CXCL8*, *IL6*, *PTGS2*, *IFIT3*, *IRF3*, *MYD88*, and *NFKB1* was detected. After 6 h of incubation with both toxins, the expression of *CCL2*, *CXCL6*, *IFIT3*, *MYD88*, *NFKB1*, and *TLR4* was higher than the controls (*P* < 0.05).

**Conclusions:**

Data indicate that in the goat MEC, LTA induces a weaker inflammatory response than LPS. This may be related to the observation that gram-positive bacteria cause chronic mastitis more often than gram-negative infections.

**Electronic supplementary material:**

The online version of this article (doi:10.1186/s40104-017-0162-8) contains supplementary material, which is available to authorized users.

## Background

Mastitis is the most prevalent disease in dairy cattle, causing the largest economic losses to the industry. The economic impact of mastitis on the U.S. dairy industry was estimated at $2 billion in 2009 [1]. The transmission of microorganisms into the mammary gland may involve the transfer of pathogens from other animals directly, from the environment or from the milking process [2]. The most common causal agent of mastitis in goats is *Staphylococcus aureus* followed by *Pasteurella haemolytica*, *Escherichia coli*, *Clostridium perfrigens*, *Streptococcus* sp., *Pseudomonas* sp., and *Nocardia* sp. [3].

Severe clinical mastitis with systemic signs produced by *S. aureus* and *E. coli* may be due to the action of various cytotoxins and endotoxins leading to extensive tissue damage and systemic reactions in the animal [2, 3]. It is well established that mastitis modifies gene expression [4, 5] and decreases animal performance [6, 7]. Toll-like receptors (TLR) play a central role in the innate immune system, and form a first line of defense against infections by recognizing pathogen associated molecular patterns [8]. In the goat, 10 TLRs have been identified, designated TLR1-TLR10 [9]. In particular, TLR2 recognizes lipoteichoic acid (LTA), a major constituent of Gram-positive bacteria, and TLR4 recognizes lipopolysaccharide (LPS) that is common to Gram-negative bacteria [8].

Innate immune responses induced by in vitro stimulation of primary mammary epithelial cells (pMEC) using LPS and LTA bacterial cell wall components are well characterized in bovine species. Numerous studies have demonstrated a potential role for TLR2 and TLR4 in the development of mastitis in dairy cattle [10], resistance to bacteria [11], and ability to affect the level of bacteria in milk [12]. Both LPS and LTA are able to cause an inflammatory response via TLR signaling [13, 14]. Activated TLR2 and TLR4 induce a common signaling pathway known as myeloid differentiation primary response 88 (MYD88)-dependent [15], and leads to the activation of kappa light polypeptide gene enhancer in B-cells 1 (NFKB1) and transcription of several pro-inflammatory genes [16].

Our hypothesis was that primary goat mammary epithelial cells (pgMEC) incubated with LPS or LTA have the capacity to mount innate immune responses that can be evaluated through changes in gene transcription. The objective of the present study was to characterize the downstream regulation of the inflammatory response induced by Toll-like receptors in pgMEC stimulated by LPS or LTA.

## Methods

### Cell culture and treatments

The pgMEC were isolated according to the method of Ogorevc and Dovč [17]. A cell culture protocol was followed involving the use of growth medium and a lactogenic medium reported in previous studies performed in bovine mammary gland cells [18]. Goat pMEC stored in liquid nitrogen were thawed and cultured in growth medium composed of MEM/EBSS (GE Healthcare, Little Chalfont, United Kingdom) supplemented with 5 mg/L insulin (Thermo Fisher Scientific, Waltham, Massachusetts), 1 mg/L hydrocortisone (Sigma-Aldrich, St. Louis, Missouri), 5 μg/mL transferrin (Sigma-Aldrich), 5 μmol/L ascorbic acid (Sigma-Aldrich), 5 mmol/L sodium acetate (Thermo Fisher Scientific), 10 mL/L penicillin/streptomycin (Sigma-Aldrich), 10% fetal bovine serum (GE Healthcare), 1 mg/L progesterone (Sigma-Aldrich), 0.05% lactalbumin (Sigma-Aldrich), 0.05% α-lactose (Sigma-Aldrich). Media were prepared daily and filtered before use with 0.22 μm Filter Unity Millex MP (EMD Millipore, Billerica, Massachusetts). Thawed cells were seeded in 25 cm^2^ flasks (10^6^ cells/flask) and cultured until confluence in 5 mL growth medium. At approximately 90% confluence, the cells were washed 3 times with 6 mL PBS (Thermo Fisher Scientific), split following the application of 3 mL 0.25% trypsin (GE Healthcare) and reseeded in new 75 mL flasks at a density of 2.5 × 10^6^ cells/flask (GE Healthcare) in 12 mL fresh growth medium. During growth and treatments the cells were incubated at 37 °C with 5% CO_2_ in Incubator KMCC17T0 (Panasonic Healthcare, Tokyo, Japan). After three passages, six 6-well plates were reseeded, 3 × 10^5^ cells/well, in 2.5 mL growth medium.

On the basis of similar studies in bovine pMEC, due to the scarcity of studies on goat cells, agonists inducing an appreciable change in TLR-related genes were selected: LPS from *Escherichia coli* O55:B5 (Sigma-Aldrich) as TLR4 agonist [19, 20] and LTA from *S. aureus* (InvivoGen, San Diego, California) as TLR2 agonist [21, 22]. The use of LPS from *E. coli* 055:B5 strain was also justified by the large number of publications demonstrating its agonist effect on TLR4 receptor in various cell types including mammary cells [20, 23, 24]. The commercial LTA preparation was prepared by the n-butanol extraction method, which preserves its activity while avoiding contamination [25].

After conducting a preliminary study, described in Additional file 1, aimed to select the incubation times and the most suitable concentrations for our purposes, the experiments were performed in 2.5 mL lactogenic medium using 1 μg/mL LPS, 20 μg/mL LTA, and the combination of both (L + L). Lactogenic C medium was composed of Dulbecco’s High Glucose Modified Eagle’s Medium (GE Healthcare) supplemented with 5 mg/L insulin (Thermo Fisher Scientific), 1 mg/L hydrocortisone (Sigma-Aldrich), 5 μg/mL transferrin (Sigma-Aldrich), 5 μmol/L ascorbic acid (Sigma-Aldrich), 5 mmol/L sodium acetate (Thermo Fisher Scientific), 10 mL/L penicillin/streptomycin (Sigma-Aldrich), 1 g/L bovine serum albumin (Sigma-Aldrich), 2.5 mg/L prolactin (Sigma-Aldrich). Triplicate cultures (1 μg/mL LPS; 20 μg/mL LTA; 1 μg/mL LPS + 20 μg/mL LTA) were performed at two incubation times (3 h, 6 h). After incubation, the cell culture supernatant was removed, cells were washed 3 times with PBS 1× and total RNA was extracted from the pgMEC layer. To check cell growth and confluence, a Light Inverted Microscope Primovert (Zeiss, Oberkochen, Germany) integrated with a high definition camera AxioCam ERc 5 s (Zeiss) was used.

### RNA extraction, purification, and quality assessment

All these procedures are described in detail in Additional file 1.

### Selection of genes, primer design, and quantitative RT-PCR

All these procedures are described in detail in Additional file 1.

### Statistical analysis

After normalization with the geometric mean of the internal control genes (*ACTB*, *GAPDH*, and *UXT*), the quantitative PCR data were log_2_-transformed before statistical analysis to obtain a normal distribution. Statistical analyses were conducted using SAS (v 9.3; SAS Institute Inc., Cary, NC). Data were analyzed using the repeated statement ANOVA with PROC MIXED. The statistical model included time (T; 3 h and 6 h incubation), treatment (TRT; LPS, LTA, LPS + LTA and control), and their interactions (T × TRT) as fixed effects. The Kenward-Roger statement was used for computing the denominator degrees of freedom, whereas spatial power was used as the covariance structure. Data were considered significant at a *P* ≤ 0.05 level using the PDIFF statement in SAS. For ease of interpretation, the expression data reported as least squares means were log_2_ back-transformed.

## Results

### Microscopy

**Fig. 1:**
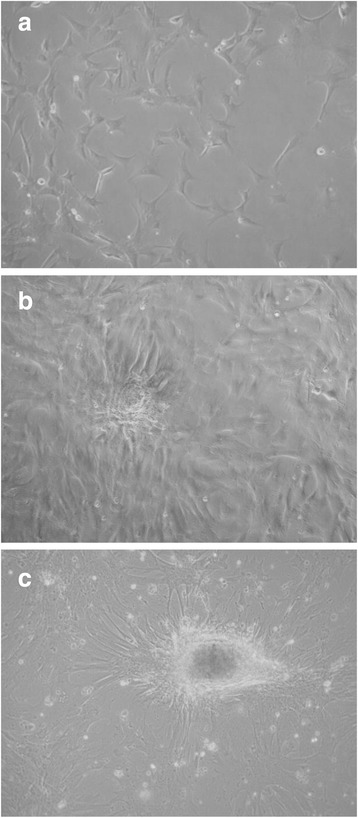
Establishment of pgMEC in culture on a collagen matrix. **a** Cobblestone-like monolayer. **b** Epithelial island. **c** Dense cellular masses

To verify the aptitude of the cells to develop typical mammary epithelial structure in culture, we carried an overgrowth experiment without harvesting the cells. During cell growth, pgMEC formed a cobblestone-like monolayer (Fig. 1a) that developed into an epithelial island within 3 d (Fig. 1b). By d 8, a central cell cluster within the epithelial islands developed into dense cellular masses (Fig. 1c). Microscopic analysis did not reveal widespread cell death or presence of cellular debris. Our observations are consistent with previous studies of cellular morphology of pMEC [19, 26, 27].

### Gene expression

The quantitative PCR performance results are reported in Table 1. Results of the statistical analyses performed on the expression profiles are in Tables 2 and 3. The expression levels of *IL1B*, *TNF* and *LTF* were deemed undetectable (>30 Ct).

**Table 1:** Quantitative PCR performance of the measured genes

Gene	Median Ct^a^	Median ∆Ct^b^	Slope^c^	(R^2^)^d^	Efficiency^e^
*CCL2*	28.62	9.66	−3.29	0.997	2.011
*CCL5*	28.95	10.05	−3.28	0.991	2.019
*CXCL6*	24.29	5.23	−3.19	0.999	2.060
*CXCL8*	29.26	10.34	−3.11	0.994	2.097
*IFIT3*	24.96	6.04	−3.07	0.993	2.117
*IL6*	29.11	10.12	−3.34	0.993	1.992
*IRF3*	24.16	5.27	−3.09	0.991	2.108
*MYD88*	24.62	5.71	−3.02	0.991	2.143
*NFKB1*	26.58	7.62	−2.91	0.996	2.204
*PTGS2*	27.47	8.49	−3.06	0.986	2.120
*TLR2*	28.51	9.64	−3.31	0.999	2.006
*TLR4*	30.20	11.28	−2.94	0.999	2.189
*TOLLIP*	23.59	4.75	−3.35	0.995	1.989

^a^The median is calculated considering all time points and treatments

^b^The median of ∆Ct is calculated as [Ct gene - geometrical mean of Ct internal controls] for each time point and treatment

^c^Slope of the standard curve

^d^R^2^ stands for the coefficient of determination of the standard curve

^e^Efficiency is calculated as [10^(−1/Slope)^]

**Table 2:** Log_2_ back-transformed LSM of gene transcription for treatment (TRT) and incubation time (T), SEM and *P* values for TRT and T

	LSM TRT^d^				LSM T		SEM		*P*-value	
Gene	Control	LPS	LTA	L + L	3 h	6 h	TRT	T	TRT	T
Cytokines										
*CCL2*	0.49^c^	1.61^a^	0.54^c^	0.80^b^	0.72	0.80	0.08	0.06	<0.0001	0.0637
*CCL5*	1.64^b^	1.58^b^	1.65^b^	1.91^a^	1.60^z^	1.78^y^	0.06	0.04	0.0022	0.0034
*CXCL6*	0.43^c^	1.58^a^	0.40^c^	0.96^b^	0.78^y^	0.66^z^	0.08	0.06	<0.0001	0.0093
*CXCL8*	0.51^c^	1.64^a^	0.43^c^	0.97^b^	0.97^y^	0.61^z^	0.12	0.09	<0.0001	<0.0001
*IL6*	1.26^b^	2.65^a^	1.27^b^	1.90^a^	1.66	1.71	0.22	0.18	0.0004	0.8208
Regulatory genes										
*IFIT3*	1.01^c^	1.13^b^	1.20^ab^	1.24^a^	0.95^z^	1.37^y^	0.04	0.03	<0.0001	<0.0001
*IRF3*	1.05^b^	1.09	1.13	1.19^a^	1.01^z^	1.22^y^	0.05	0.04	0.0818	<0.0001
*MYD88*	1.73^b^	2.05^a^	1.81^b^	2.11^a^	1.81^z^	2.03^y^	0.03	0.03	<0.0001	<0.0001
*NFKB1*	1.13^c^	1.49^a^	1.34^b^	1.51^a^	1.10^z^	1.68^y^	0.05	0.04	<0.0001	<0.0001
*PTGS2*	1.05^b^	1.30^a^	1.32^a^	1.29^a^	1.03^z^	1.48^y^	0.05	0.04	<0.0001	<0.0001
*TLR2*	10.39	11.38	10.11	11.24	10.58	10.96	0.09	0.07	0.3747	0.5420
*TLR4*	1.04^c^	1.49^a^	1.04^c^	1.22^b^	1.18	1.19	0.08	0.06	<0.0001	0.8645
*TOLLIP*	0.96^b^	0.96^b^	1.04^a^	0.95^b^	0.98	0.97	0.02	0.01	<0.0001	0.3915

^a-c^Different letters represent significant differences between treatments (*P* < 0.05)

The letter a indicates higher transcript levels than b and c. The letter b indicates higher transcript levels than c

^d^Treatments: Control = incubation without toxins; LPS = incubation with 1 μg/mL lipopolysaccharide; LTA = incubation with 20 μg/mL lipoteichoic acid; L + L = incubation with the combination of both toxins

^y-z^Different letters represent significant differences between time points (*P* < 0.05). The letter y indicates higher transcript levels than z

**Table 3:** Log_2_ back-transformed LSM of interactions between treatment (TRT) and incubation time (T) on gene transcription, SEM and *P* values for TRT × T

		LSM TRT^d^ × T				SEM	*P* value
Gene	T	Control	LPS	LTA	L + L	TRT × T	TRT × T
Cytokines							
*CCL2*	3 h	0.45^c^	1.83^a,y^	0.48^c,z^	0.69^b,z^	0.11	0.0040
	6 h	0.53^c^	1.41^a,z^	0.61^c,y^	0.94^b,y^		
*CCL5*	3 h	1.40^c,z^	1.45^bc,z^	1.62^b^	2.00^a^	0.07	0.0018
	6 h	1.91^y^	1.72^y^	1.68	1.83		
*CXCL6*	3 h	0.43^c^	1.67^a^	0.51^c,y^	0.99^b^	0.12	0.0274
	6 h	0.44^c^	1.49^a^	0.32^d,z^	0.93^b^		
*CXCL8*	3 h	0.49^c^	2.17^a,y^	0.62^c,y^	1.34^b,y^	0.17	0.0085
	6 h	0.52^b^	1.24^a,z^	0.30^c,z^	0.70^b,z^		
*IL6*	3 h	1.01^b^	3.01^a^	1.15^b^	2.17^a^	0.28	0.1423
	6 h	1.57	2.33	1.41	1.66		
Regulatory genes							
*IFIT3*	3 h	0.85^b,z^	0.96^a,z^	0.96^a,z^	1.05^a,z^	0.05	0.5151
	6 h	1.19^b,y^	1.34^a,y^	1.48^a,y^	1.47^a,y^		
*IRF3*	3 h	0.94^b,z^	1.01^z^	0.99^z^	1.12^a^	0.07	0.3942
	6 h	1.18^y^	1.18^y^	1.28^y^	1.26		
*MYD88*	3 h	1.64^b,z^	1.95^a,z^	1.67^b,z^	2.02^a^	0.05	0.7335
	6 h	1.82^b,y^	2.16^a,y^	1.96^b,y^	2.20^a^		
*NFKB1*	3 h	0.93^b,z^	1.18^a,z^	1.12^a,z^	1.21^a,z^	0.06	0.5318
	6 h	1.37^c,y^	1.88^a,y^	1.62^b,y^	1.90^a,y^		
*PTGS2*	3 h	0.85^b,z^	1.12^a,z^	1.07^a,z^	1.12^a,z^	0.07	0.2535
	6 h	1.31^b,y^	1.50^y^	1.63^a,y^	1.48^y^		
*TLR2*	3 h	9.46	10.76	10.80	11.41	0.12	0.2028
	6 h	11.41	12.04	9.47	11.07		
*TLR4*	3 h	0.99^c^	1.42^a^	1.28^ab,y^	1.08^bc,z^	0.10	<0.0001
	6 h	1.09^b^	1.57^a^	0.85^c,z^	1.37^a,y^		
*TOLLIP*	3 h	0.96^b^	0.97^b^	1.03^a^	0.97^b^	0.03	0.3689
	6 h	0.96^b^	0.94^b^	1.06^a^	0.94^b^		

^a-c^Different letters represent significant differences between treatments within the same incubation time (*P* < 0.05). The letter a indicates higher transcript levels than b and c. The letter b indicates higher transcript levels than c

^d^Treatments: LPS = incubation with 1 μg/mL lipopolysaccharide; LTA = incubation with 20 μg/mL lipoteichoic acid; L + L = incubation with the combination of both toxins; Control = incubation without toxins

^y-z^Different letters represent significant differences between time points within the same treatment (*P* < 0.05). The letter y indicates higher transcript levels than z

#### Chemokines and interleukins

We observed a treatment effect for *CCL2* (*P* < 0.0001), *CCL5* (*P* < 0.003), *CXCL6* (*P* < 0.0001), *CXCL8* (*P* < 0.0001), and *IL6* (*P* < 0.001) (Table 2). Incubation time affected *CCL5* (*P* < 0.004), *CXCL6* (*P* < 0.01) and *CXCL8* genes (*P* < 0.0001) (Table 2). Several significant differences (*P* < 0.05) were found for the interactions between treatment and time (Table 3). Details on these differences are illustrated as follows.

There was an overall increase in most transcript levels in the presence of LPS (*P* < 0.0001), and both toxins (*P* < 0.001) with respects to controls. *CCL2* transcription was higher in response to both toxins vs. LTA alone (*P* < 0.01). The combination of both toxins decreased (*P* < 0.001) CCL2 transcription compared to incubation with LPS alone. The highest transcript expression occurred in samples incubated for 3 h in the presence of LPS (*P* < 0.0001). Compared to 3 h, at 6 h incubation the *CCL2* transcription was relatively higher in response to LTA (*P* < 0.05) and both toxins (*P* < 0.01), but was lower in the presence of LPS alone (*P* < 0.03).

After 3 h, *CCL5* transcript levels increased in samples incubated with both toxins compared to LPS alone (*P* < 0.0001), LTA alone (*P* < 0.005) and control samples (*P* < 0.0001). Incubation for 3 h with LTA alone increased *CCL5* transcription with respect to controls (*P* < 0.05). Although no time effect was detected at 3 h for *CCL5* regardless of treatment, after 6 h the expression of *CCL5* increased with LPS alone (*P* < 0.02) and in the controls (*P* < 0.0001).

After 3 and 6 h, treatments with LPS alone or in combination with LTA increased *CXCL6* transcription (*P* < 0.0001) when compared to controls and LTA alone. At 3 h (*P* < 0.0001) and 6 h (*P* < 0.001) of incubation, LPS alone increased *CXCL6* transcription compared to the incubation with both toxins. A time dependent effect was detected only in samples incubated with LTA, with a decrease of expression in samples incubated for 6 vs. 3 h (*P* < 0.001). After 3 h, the *CXCL8* transcription was higher in LPS samples vs. controls (*P* < 0.0001), LTA alone (*P* < 0.0001) and both toxins (*P* < 0.01). After 6 h, transcription was higher in controls vs. LTA alone (*P* < 0.01) but lower in controls vs. LPS alone (*P* < 0.0001). Furthermore, after 6 h *CXCL8* transcription was higher for LPS alone compared to LTA alone (*P* < 0.0001), both toxins vs. LTA alone (*P* < 0.0001), or LPS alone vs. both toxins (*P* < 0.002). Although no time effect was detected at 3 h for *CXCL8* regardless of treatment, after 6 h, the expression of *CXCL8* decreased with LPS alone (*P* < 0.002), LTA alone (*P* < 0.0001) and both toxins (*P* < 0.001).

Incubation for 3 h with both toxins increased *IL6* transcription vs. controls (*P* < 0.005) and LTA alone (*P* < 0.02). After 3 h incubation, LPS alone increased *IL6* transcript levels compared to controls and LTA alone (*P* < 0.001).

#### Other regulatory genes

A treatment effect (*P* < 0.0001) was detected for transcription of *IFIT3*, *MYD88*, *NFKB1*, *PTGS2*, *TLR4* and *TOLLIP* whereas incubation time affected *IFIT3*, *IRF3*, *MYD88*, *NFKB1* and *PTGS2* transcription (*P* < 0.0001) (Table 2). Several significant differences (*P* < 0.05) occurred for the interactions between treatment and incubation time (Table 3). Details on these differences are illustrated below.

After 3 h, *IFIT3* transcript levels were lower in controls vs. LPS (*P* < 0.04), LTA (*P* < 0.03) and both (*P* < 0.001). The same trend occurred after 6 h when *IFIT3* transcription was lower in controls vs. LPS (*P* < 0.04), LTA (*P* < 0.001) and both (*P* < 0.001). Incubation (6 h vs. 3 h) always increased (*P* < 0.0001) *IFIT3* transcript levels. We found higher *IRF3* transcript levels in samples incubated with both toxins vs. controls (*P* < 0.01) after 3 h incubation. A time dependent increase occurred for LPS (*P* < 0.03), LTA (*P* < 0.001) and controls (*P* < 0.002).

After 3 h, *MYD88* transcript levels were lower in controls than LPS (*P* < 0.001) or both toxins (*P* < 0.0001), whereas LTA generated lower transcript levels than LPS alone (*P* < 0.003) or in combination with LTA (*P* < 0.001). After 6 h, *MYD88* transcript levels were lower in controls than LPS (*P* < 0.001) or both toxins (*P* < 0.001), whereas LTA generated lower *MYD88* transcript levels than LPS alone (*P* < 0.05) or in combination with LTA (*P* < 0.02). Incubation increased *MYD88* transcription in samples with LPS (*P* < 0.04), LTA (*P* < 0.003) and controls (*P* < 0.04).

Incubation increased *NFKB1* transcription in all samples (*P* < 0.0001). After 3 h, *NFKB1* transcript levels were lower in controls than LPS (*P* < 0.001), LTA (*P* < 0.002) and both (*P* < 0.0001). After 6 h, *NFKB1* transcription was lower in controls than LPS (*P* < 0.0001), LTA (*P* < 0.01) and both (*P* < 0.0001). Furthermore, at 6 h incubation, transcription was lower in LTA vs. LPS (*P* < 0.01) and both toxins (*P* < 0.01).

After 3 h *PTGS2* transcript levels were lower in controls vs. LPS (*P* < 0.001), LTA (*P* < 0.002) and both toxins (*P* < 0.001). After 6 h only LTA increased *PTGS2* transcript levels vs. controls (*P* < 0.004). Incubation always increased *PTGS2* transcription, i.e. LPS (*P* < 0.0001), LTA (*P* < 0.0001), both toxins (*P* < 0.001) and controls (*P* < 0.0001).

After 3 h, *TLR4* transcript levels were lower in controls than in the presence of LTA (*P* < 0.01) and LPS (*P* < 0.001). Moreover, *TLR4* transcription was higher in samples incubated with LPS vs. both toxins (P < 0.005). After 6 h, *TLR4* transcript levels were lower in LTA samples vs. controls (P < 0.01), LPS (P < 0.0001) and both toxins (*P* < 0.0001), in controls vs. LPS (*P* < 0.001) and both toxins (*P* < 0.02). A time dependent increase was found in samples incubated with both toxins (*P* < 0.02) whereas a time dependent decrease occurred for LTA (*P* < 0.0001).

After 3 h, *TOLLIP* transcript levels were significantly higher in samples incubated with LTA vs. controls (*P* < 0.02), LPS (*P* < 0.03) and both toxins (*P* < 0.03). After 6 h *TOLLIP* transcription was also higher for LTA vs. controls (*P* < 0.001), LPS (*P* < 0.001) and both toxins (*P* < 0.0001). No significant difference was found among treatments and time points in *TLR2* transcription levels.

## Discussion

### Chemokines and interleukins

Chemokines regulate migration and adhesion of infiltrating cells to an inflamed lesion [28], and inhibition of chemokine expression or secretion significantly reduces cell infiltration [29]. Resident tissue cells such as mesangial cells and inflammatory cells such as monocytes/macrophages stimulate expression and secretion of chemokines [30]. The chemokines *CCL2* and *CCL5*, which belong to the “type I IFN chemokine signature”, attract mainly monocytes, natural killer cells and activated lymphocytes [31, 32]. Thus, interferon (IFN) signaling is considered a critical point for host resistance against different pathogens [33], although the end result may be beneficial or detrimental to the host depending on the circumstances [34]. As reported previously in non-ruminants [35], the differential expression of these IFN-regulated chemokines with LPS or LTA could indicate a stronger recruitment of monocytes and lymphocytes in the mammary tissue and milk.

The greater expression of *CCL2* with LPS than LTA was consistent with data from a study with bovine pMEC incubated with LPS purified from *E. coli* strain O55:B5 [19, 20] or heat-inactivated *E. coli* [36], and the lack of effect of LTA isolated from *Streptococcus pyogenes* [19], *S. aureus* [20] or heat-inactivated *S. aureus* [36]. The down-regulation of *CCL2* with L + L than LPS might have been due to an interaction between LPS and LTA. Recent work has led to the speculation that bifidobacteria could induce cross-tolerance in bovine intestinal epithelial cells through their interaction with TLR2 [37]. In addition, it has been speculated that pre-exposure to LTA and lipopeptides which trigger TLR2-mediated signaling led to tolerance to LPS [38]. The lack of LPS effect on *CCL5* is in contradiction to a similar study with bovine MEC using 20 μg/mL LPS from *E. coli* O55:B5 [20]. This discrepancy might be explained by the different concentrations used in the studies.

The chemokines *CCL2* and *CXCL6* have strong chemo-attractant activities [39]. The up-regulation of *CXCL6* with LPS is similar to a previous study where *CCL2* and *CXCL6* increased markedly upon LPS challenge of MEC [19]. Mastitis is strongly associated with increased somatic cell counts in milk, the majority of which is attributable to neutrophils and lymphocytes [40]. Local production of pro-inflammatory cytokines in mammary tissue may have a strong influence on the activation state of the infiltrating neutrophils [41].

The temporal response in *CXCL8* after 3 and 6 h in the presence of LPS is similar to results reported in a previous study incubating bovine MEC with 50 μg/mL LPS or 20 μg/mL LTA, where an initial increase of *CXCL8* transcript levels after 2 h was followed by a decrease after 4 h in the presence of LTA and LPS [19]. In addition, a similar trend has been detected in a study performed with endometrial epithelial cells incubated with LPS where *CXCL8* levels were higher after 3 h incubation vs. 6 h [23].

The cytokine *IL6* is a pleiotropic protein with a strong influence on inflammatory responses, and is a major effector of the acute-phase reaction [42]. Thus, the observation that LPS alone or in combination with LTA up-regulated *IL6* only after 3 h could be explained by its quick mechanism of action, which was also reported previously in bovine MEC [20].

### Other regulatory genes

The up-regulation of *IFIT3* with LPS alone compared to controls at 3 and 6 h is consistent with a previous study with bovine MEC using 20 μg/mL LPS from *E. coli* O55:B5 [20]. Activation of TLR4 by LPS induces the MyD88-independent pathway that promotes the internalization of the antigen-receptor LPS-TLR4 complex and activates interferon regulatory factor 3 (*IRF3*) [43]. The observed up-regulation of *IFIT3* with LTA might have been due to the responsiveness of this gene to a large variety of exogenous molecules [44]. The induction of the interferon induced protein with tetratricopeptide repeats (IFIT gene family) by different stimuli is based on the activation of interferon regulatory factors, which recognize the IFN-stimulated response elements (ISRE) in the IFIT promoters and initiate transcription [45].

IRF3 is involved in the MyD88-independent signaling pathway activated by TLR4, which may explain the lack of effect detected in *IRF3* between LTA alone and controls. However, the lack of an increase in *IRF3* transcription with LPS alone was unexpected because *IRF3* should be activated by TLR4 [43]. In a previous study with bovine mammary epithelial cells (MAC-T) [46], no significant *IRF3* increase was detected until 6 h incubation with 1 μg/mL LPS from *E. coli* J5 Rc mutant. The increase in *IRF3* transcription at 3 h incubation with both toxins could be explained by an interaction effect between LPS and LTA on pgMEC.

The published data regarding *MYD88* regulation induced by LPS or LTA are seemingly discordant. For example, a non-significant down-regulation of *MYD88* has been observed after 24 h with 50 μg/mL LPS treatment in immortalized bovine MEC, with no differences detected in primary bovine MEC [19]. In a study performed with immortalized bovine MEC [46], LPS induced the up-regulation of adaptor *MYD88* transcript that increased gradually compared to untreated cells and peaked significantly at 72 h after induction. In endometrial epithelial cells, *MYD88* expression peaks at 6 h after LPS-treatment [23]. Our data were more consistent with a study performed in endometrial stromal cells and whole endometrial cells incubated with LPS and LTA [47]. In that study, LPS stimulation up-regulated *MYD88* expression after 8 h in both cell types, whereas LTA stimulation of whole endometrial cells was associated with a non-significant increase of *MyD88*. Thus, it appears that a positive feedback loop with TLR4-dependent molecular self-regulation of the downstream signaling MyD88 [48] could partly explain our data.

The up-regulation of *NFKB1* with all challenges was consistent with previous studies where bacterial infections up-regulated *NFKB1* transcription in bovine mammary cells, confirming the ability of the mammary gland to mount a robust innate immune response [41, 46, 49]. Furthermore, our data agree with a previous study reporting up-regulation of *NFKB1* in bovine endometrial epithelial cells challenged with LPS [23].

Prostaglandins are one of several inflammatory mediators in the bovine mammary gland with chemotactic activity [50], hence, explaining the up-regulation of *PTGS2* with all challenges after 3 h. The PTGS2 protein is one of the enzymes involved in prostaglandin synthesis that is transiently up-regulated during inflammation [51]. *PTGS2* expression is increased by LTA [52]. The induction of *PTGS2* could have been associated with the action of MyD88 and activation of NFκB as reported previously [53].

The lack of effect on *TLR2* expression in the present study is consistent with a previous study of bovine MEC after 6 h incubation with heat-inactivated *E. coli* or after 30 h incubation with heat-inactivated *S. aureus* [36]. However, both datasets contrast the significant up-regulation of *TLR2* induced by LPS or heat-killed *E. coli* treatment of bovine endometrial cells for 3 and 6 h [23]. It could be possible that LTA inhibited TLR signaling as reported previously in human monocyte-like cells [54].

The greater *TLR4* expression due to LPS when compared to controls is consistent with previous data from a study performed with bovine MEC where *TLR4* was greater than controls in cells incubated for 6 h with 1 μg/mL LPS from *E. coli* [46]. Similar to the decrease that we detected over time for *TLR4* upon LTA challenge, the expression of *TLR4* had decreased in endometrial epithelial cells incubated for 3 and 6 h with 100 μg/mL LPS from *E. coli* after a significant increase at 1 h incubation [23].

The lower *CXCL6* and *CXCL8* expression after 3 and 6 h incubation induced only by LTA coincided with the higher expression of *TOLLIP* (Table 3), which is consistent with its anti-inflammatory role [55–57]. A time-dependent increase in *TOLLIP* has been reported in bovine MEC incubated with 1.0 μg/mL LPS from *E. coli* mutant J5 for 24 h; whereas a time-dependent decrease had occurred between 48 and 72 h of incubation [46]. These data indicate that an up-regulation of *TOLLIP* is necessary to counteract the harmful effects associated with over production of cytokines. In fact, using short hairpin RNA knockdown of TOLLIP in peripheral blood human monocytes, TOLLIP suppresses TNF and IL-6 production after stimulation with TLR2 and TLR4 agonists, and induces secretion of the anti-inflammatory cytokine IL-10 [58].

## Conclusions

Consistent with numerous experiments in bovine mammary epithelial cells, our study confirms the capacity of LPS to stimulate inflammatory genes acting as TLR4 agonists in pgMEC. The differences in gene expression responses of goat mammary epithelial cells to LPS and LTA revealed different activation pathways for these components of Gram-negative and Gram-positive bacterial cell walls. Further studies focused on protein expression changes should be carried out to confirm gene transcription variation at the translation level. Furthermore, genes and corresponding proteins involved in cellular apoptosis should be studied in order to investigate potential mechanisms damaging goat mammary tissue in response to inflammatory stimuli. The challenge with LPS compared to LTA generated much stronger and sustained responses that seem to reflect an adaptation to the more acute nature of mastitis caused by coliform bacteria. The lack of response for some pro-inflammatory cytokines during incubation with LTA indicates some degree of tolerance to this agent, consistent with chronic infections of the mammary tissue caused by *Staphylococcal* species.

## Additional file

Additional file 1: Additional materials. RNA extraction, purification, and quality assessment; selection of genes, primer design, quantitative RT-PCR, **Table S1.** Genes analyzed by quantitative PCR, and **Table S2.** Oligonucleotide primer sequences. (DOCX 31 kb)

## Abbreviations

ACTB: Actin beta
CCL2: C-C motif chemokine ligand 2
CCL5: C-C motif chemokine ligand 5
CXCL6: C-X-C motif chemokine ligand 6
CXCL8: Interleukin 8
GAPDH: Glyceraldehyde-3-phosphate dehydrogenase
IFIT: Interferon induced protein with tetratricopeptide repeats
IFIT3: Interferon induced protein with tetratricopeptide repeats 3
IFN: Interferon
IL1B: Interleukin 1 beta
IL6: Interleukin 6
IRF3: Interferon regulatory factor 3
ISRE: IFN-stimulated response elements
LPS: Gram-negative lipopolysaccharide
LTA: Gram-positive lipoteichoic acid
LTF: Lactoferrin
MEC: Mammary epithelial cells
MYD88: Myeloid differentiation primary response 88
NFKB1: Nuclear factor of kappa light polypeptide gene enhancer in B-cells 1
pgMEC: Primary goat mammary epithelial cells
pMEC: Primary mammary epithelial cells
PTGS2: Prostaglandin-endoperoxide synthase 2
qPCR: Quantitative real-time PCR
T: Time
TLR: Toll-like receptors
TLR1-TLR10: Toll-like receptors 1–10
TLR2: Toll-like receptor 2
TLR4: Toll-like receptor 4
TNF: Tumor necrosis factor alpha
TOLLIP: Toll interacting protein
TRT: Treatment
UXT: Ubiquitously expressed prefoldin like chaperone
