# Supplementary material for: Innate immune responses induced by lipopolysaccharide and lipoteichoic acid in primary goat mammary epithelial cells
Source: J Anim Sci Biotechnol. 2017 Apr 1;8:29. doi: 10.1186/s40104-017-0162-8 (PMC5379715; doi:10.1186/s40104-017-0162-8)
Supplement: Additional file 1: — Additional materials. RNA extraction, purification, and quality assessment; selection of genes, primer design, quantitative RT-PCR, Table S1. Genes analyzed by quantitative PCR, and Table S2. Oligonucleotide primer sequences. (DOCX 31 kb) [file 40104_2017_162_MOESM1_ESM.docx]

**Supplemental Materials**

**Total RNA extraction, purification and quality assessment**

Total RNA was extracted incubating 1 minute at room temperature with 1 mL/well QIAzol Lysis Reagent (QIAGEN, Hilden, Germany), collected in 2 mL tubes and frozen at -80°C overnight. The day after, RNA was thawed at room temperature, then kept on ice during the first steps of purification. Briefly, after thawing, samples were at room temperature for 5 min, and 200 μL Chloroform (Thermo Fisher Scientific) were added prior to mixing vigorously for 15 s. After 3 min at room temperature samples were centrifuged 15 min at 12000 × g at 4°C. The upper phase was transferred into a new collection tube without disturbing the mid and lower phases while on ice.

Working at room temperature, 750 μL 100% Ethanol (Decon Labs) were added and mixed thoroughly by pipetting several times. To purify the samples a miRNeasy Mini Kit (QIAGEN) was used following the handbook furnished with the kit. The samples were loaded to the miRNeasy mini spin column and centrifuged 30 s at 12000 rpm discarding the flow through. To wash the column, 350 μl RWT buffer were added and centrifuged 30 s at 12000 rpm discarding the flow through. To eliminate every DNA residue, columns were incubated 15 min at room temperature with 80 μL DNase I (RNase-Free DNase Set, QIAGEN), 350 μL RWT buffer were added into the miRNeasy mini spin column, then centrifuged for 30 s at 12000 rpm discarding the flow through. The column was washed with 500 μL RPE buffer and centrifuged 30 s at 12000 rpm. The washing step was repeated with 500 μL RPE centrifuging for 2 min at 12000 rpm. The membrane was dried by centrifuging 2 min at 15000 rpm.

The RNA was eluted with 50 μL DNase/RNase free water by centrifuging 1 min at 12000 rpm and stored at -80°C until use. The RNA quantification was performed using the NanoDrop ND-1000 spectrophotometer (Thermo Fisher Scientific), RNA purity average (A_260_/A_280_) was >2. The RNA Integrity Number assessed using RNA Nano Chips (Agilent Technologies, Santa Clara, California) and Bionalyzer (Agilent Technologies) 2100 was >9. A portion of the samples was diluted to 100 ng/μL in DNase/RNase free water for reverse transcription into cDNA.

**Selection of genes and primer design**

The choice of genes for qPCR was based on their role in the inflammatory response, chemotaxis, acute-phase of inflammation, antimicrobial activity, and downstream targets of TLR signaling and innate immune response (Table 1S). Three verified internal control genes (*ACTB*, *GAPDH* and *UXT*) were also selected according to a previous work on bovine cells [1].

Primer pairs for all genes were designed using publicly available goat sequences. Database research, alignments, and sequence analyses were performed with the aid of NCBI (<http://www.ncbi.nlm.nih.gov/>). Goat genes were run in BLASTN to check identity and orthology. Primer design and optimization were carried out using Primer3 and BLAST (<http://www.ncbi.nlm.nih.gov/tools/primer-blast/>). The primers selected had a product size ranging from 100 to 200 bp, melting temperatures between 57 and 63°C and, if possible, were separated by at least one intron and spanned an exon-exon junction. The referred organism utilized was *Capra hircus* (taxid:9925). All the primers in Table 2S were ordered from Integrated DNA Technologies (Coralville, Iowa). Primers reconstituted were diluted to have a 10 μ*M* work solution ready to use.

**Reverse Transcription**

Sufficient cDNA was prepared to run all selected genes and synthesized by reverse transcription PCR using a Mastercycler Gradient (Eppendorf, Hamburg, Germany) keeping the lid during the reaction at 70°C. To 1 μL 100 ng/μL RNA we added 1 μL Random Primers (Roche, Basel, Switzerland) and 9 μL DNase/RNase free water. The mixture was incubated at 65°C for 5 min and kept on ice for 3 min. To this mixture we added 9 μL mater mix composed by 1.625 μL DNase/RNase free water, 4 μL Reaction Buffer 5 X for RT (Thermo Fisher Scientific), 1 μL Oligo dT18 (Integrated DNA Technologies), 2 μL mmol/L dNTP mix 10 mmol/L (Invitrogen, Carlsbad, California), 0.125 μL Ribolock RNase Inhibitor (Thermo Fisher Scientific), 0.25 μL RevertAid RT (Thermo Fisher Scientific) and was incubated for 5 min at 25°C, 1 h at 42°C and 5 min at 70°C to synthesize cDNA. A portion of cDNA was mixed to make a pool to test primers using PCR, and a portion was diluted 1:4 with DNase/RNase free to run quantitative PCR.

**Primer Test**

Each primer pair was tested by PCR to analyze the primer specificity. We mixed 8 μL cDNA diluted 1:4 in DNase/RNase free water cDNA, 10 μL PerfeCTa® SYBR® Green FastMix®, ROX™ (Quantabio, Gaithersburg, Maryland) and 1 μL of each 10 μmol/L primer that compose the specific pair. To perform this reaction we used a Mastercycler Gradient (Eppendorf) keeping the lid at 100°C. After 2 min at 50°C and 10 min at 95°C we repeated 41 cycles composed from 15 s at 95°C and a minute at 60°C. We checked amplification with a electrophoretic run (OWL-OSP-105-Electrophoresis-Power-Supply-120V-60-Hz, Thermo Fisher Scientific) in 2% Low EEO Agarose (Thermo Fisher Scientific) dissolved in TAE buffer (Thermo Fisher Scientific) with 15 μl SYBR Safe DNA gel Stain (Invitrogen). After a constant voltage run of 100 volts, the gel was acquired with the ChemiDoc™ MP System (Bio-Rad, Hercules, California) using ultraviolet light emitted from a transilluminator.

**Quantitative Real-Time PCR**

Quantitative Real-Time PCR (Q-PCR) was performed combining 4 μL diluted 1:4 cDNA with 5 μL PerfeCTa® SYBR® Green FastMix®, ROX™ (Quantabio), 0.4 μL of each forward and reverse primer, and 0.2 μL of DNase/RNase free water in a MicroAmp Optical 384-Well Reaction Plate (Applied Biosystems, Foster City, California). Each sample was run in duplicate and a 7 point-standard curve plus the non-template control in triplicate. The reactions were performed in a 7900HT Fast Real-Time PCR System (Applied Biosystems) using the following conditions: a first denaturation step of 5 min at 95°C, a second amplification step composed of 40 cycles of 1 second at 95°C (denaturation) and 30 s at 60°C (annealing and extension), a third dissociation step composed of 15 s at 95°C, 15 s at 60°C and 15 s at 95°C to verify the presence of a single PCR product. Data were calculated with the ABI 7900HT Sequence Detection Systems Version 2.4 (Applied Biosystems). The coefficients of determination of the standard curve for each gene were higher than 0,99 for all genes except for *PTGS2* (R^2^ = 0.986). The final data were normalized using the geometric mean of the 3 housekeeping genes [1].

**Preliminary study**

A preliminary study was performed to select the incubation times and the most suitable concentration for our purposes. The choice of time points and concentrations utilized was carried out after experiments conducted with three incubation times and three concentrations of toxins. Goat pMEC were incubated for 3, 6 and 12 h in presence of 1, 10 and 20 μg/mL toxins (LPS, LTA and the combination of both). After RNA extraction, the RNA quantification and the RNA Integrity Number were assessed. Transcription levels of 10 genes (*ACTB*, *CXCL6*, *CXCL8*, *IL1B*, *IL6*, *GADPH*, *TLR2*, *TLR4*, *TNF* and *UXT*) were measured by qPCR to check the responsiveness of our cells to the toxins selected.

**Table S1** Genes analyzed by quantitative PCR

| Gene | Function | Class | References^1^ |
| --- | --- | --- | --- |
| Cytokines | | | |
| *CCL2* | Recruiting monocytes, memory T cells, dendritic cells | Chemokine | 2 |
| *CCL5* | Chemotactic for T cells eosinophils and basophils | Chemokine | 3 |
| *CXCL6* | Chemoattractant for neutrophilic granulocytes | Chemokine | 2 |
| *CXCL8* | Chemoattractant activity | Interleukin | 2 |
| *IL1B* | Lymphocyte activating | Interleukin | 2, 4 |
| *IL6* | Pro-inflammatory and anti-inlfammatory | Interleukin | 4 |
| *TNF* | Acute phase reaction | Cytokine inflammatory | 2, 4 |
| Other regulatory genes | | | |
| *IFIT3* | Interferon-induced protein | IFN-related genes | 3 |
| *IRF3* | Activating transcription of interferons alpha and beta | Transcription Regulatory | 5 |
| *LTF* | Antimicrobial activity (sequestering iron) | Antimicrobial | 4 |
| *MYD88* | Interaction with TLR4 | Adaptor protein | 2, 5 |
| *NFKB1* | Regulating the immune response to infection | Transcription Regulatory | 2, 5 |
| *PTGS2* | Conversion of arachidonic acid to prostaglandin H2 | Cytochrome oxidase | 4 |
| *TLR2* | Innate Immune System | Membrane receptor | 2, 5 |
| *TLR4* | Innate Immune System | Membrane receptor | 2, 5 |
| *TOLLIP* | Interacting with TLR | Adaptor protein | 5 |
| Housekeeping genes | | | |
| *ACTB* | Cell motility, structure and integrity | Cytoskeletal | 2, 5 |
| *GAPDH* | Transcription activation (initiation of apoptosis) | Regulatory protein | 2, 4 |
| *UXT* | Receptor induced transcriptional activation | Transcript protein | 5 |

**^1^**References are reported where gene transcription was found increased in similar studies on bovine cells.

**Table S2** Oligonucleotide primer sequences

| Gene | Forward | Reverse | Reference Sequence^1^ |
| --- | --- | --- | --- |
| *ACTB* | CGTGATGGTGGGCATGGG | GGTGTGGTGCCAGATCTTCT | NM_001314342 |
| *CCL2* | CTCGCTCAGCCAGATGCAAT | AGGTTGGGGTCTGCACAAAA | XM_005693218 |
| *CCL5* | GTCTGCCTCCCCATATGCCTC | CTCTCGCACCCACTTCTTCTC | XM_005693201 |
| *CXCL6* | CCAAGGTGGAAGTGGTAGCC | CTGGGCAATTCTTCCAACGC | XM_005681937 |
| *CXCL8* | TGTGTGAAGCTGCAGTTCTGT | TGGGGTCTAAGCACACCTCT | XM_005681749 |
| *GAPDH* | TGGTGAAGGTCGGAGTGAAC | TGTAGACCATGTAGTGAAGGTCA | XM_005680968 |
| *IFIT3* | AAATTCTGAGGCAGGCCGTT | TTTCCCAGAGCCTCGACAAC | XM_005698196 |
| *IL1B* | TCCACCTCCTCTCACAGGAAA | TACCCAAGGCCACAGGAATCT | XM_013967700 |
| *IL6* | TCTGGGTTCAATCAGGCGAT | TGTTTGTGGCTGGAGTGGTT | NM_001285640 |
| *IRF3* | TTGTGAACTCAGGGGTCAGG | TGGGCTCAAGTCCATGTCAC | XM_013971473 |
| *LTF* | GCAGAGAACCGGAAATCCTC | AAGTGAGCCCCTCATTTGCT | NM_001285548 |
| *MYD88* | TTGAGAAGAGGTGCCGTCG | CAGACAGTGATGAAGCGCAG | XM_013973392 |
| *NFKB1* | CTGGAAGCACGAATGACAGA | GCTGTAAACATGAGCCGTACC | XM_005681365 |
| *PTGS2* | CTCTGCGGTGCAGCAAATC | ATGTAGTGCACTGTGTCGGG | XM_005690989 |
| *TLR2* | TTGACAAGAAGGCCATCCCC | AGAACGCTTCCTGCTGAGTC | NM_001285603 |
| *TLR4* | TTCAACCGTATCACGGCCTC | TGACCCACTGCAGGAAACTC | NM_001285574 |
| *TNF* | GCACTTCGGGGTAATCGGC | GCCTTGAGGGCATTGGCAT | NM_001286442 |
| *TOLLIP* | CGACGTAGGCTTAGCGTGAA | CTGGTCTCACGCATCTACCG | XM_013976999 |
| *UXT* | TGTGGCCCTTGGATATGGTT | GGTTGTCGCTGAGCTCTGTG | XM_005700842 |

^1^GenBank Accession Number

**References**

1. Bionaz M, Loor JJ. Identification of reference genes for quantitative real-time PCR in the bovine mammary gland during the lactation cycle. Physiol Genom. 2007;29:312-9.
2. Strandberg Y, Gray C, Vuocolo T, Donaldson L, Broadway M, Tellam R. Lipopolysaccharide and lipoteichoic acid induce different innate immune responses in bovine mammary epithelial cells. Cytokine. 2005;31:72-86.
3. Gilbert FB, Cunha P, Jensen K, Glass EJ, Foucras G, Robert-Granie C, et al. Differential response of bovine mammary epithelial cells to *Staphylococcus aureus* or *Escherichia coli* agonists of the innate immune system. Vet Res. 2013;44:40.
4. Zbinden C, Stephan R, Johler S, Borel N, Bunter J, Bruckmaier RM, et al. The inflammatory response of primary bovine mammary epithelial cells to *Staphylococcus aureus* strains is linked to the bacterial phenotype. PLoS One. 2014;9:e87374.
5. Ibeagha-Awemu EM, Lee JW, Ibeagha AE, Bannerman DD, Paape MJ, Zhao X. Bacterial lipopolysaccharide induces increased expression of toll-like receptor (TLR) 4 and downstream TLR signaling molecules in bovine mammary epithelial cells. Vet Res. 2008;39:11.
